# Approaches to identify and characterize microProteins and their potential uses in biotechnology

**DOI:** 10.1007/s00018-018-2818-8

**Published:** 2018-04-18

**Authors:** Kaushal Kumar Bhati, Anko Blaakmeer, Esther Botterweg Paredes, Ulla Dolde, Tenai Eguen, Shin-Young Hong, Vandasue Rodrigues, Daniel Straub, Bin Sun, Stephan Wenkel

**Affiliations:** 1 Department of Plant and Environmental Sciences, University of Copenhagen, Thorvaldsensvej 40, 1871 Frederiksberg C, Denmark; 2 Copenhagen Plant Science Centre, University of Copenhagen, Thorvaldsensvej 40, 1871 Frederiksberg C, Denmark

**Keywords:** MicroProtein, Small proteins, Targets, Complex, MiPFinder, Inhibition, Protein–protein interaction

## Abstract

MicroProteins are small proteins that contain a single protein domain and are related to larger, often multi-domain proteins. At the molecular level, microProteins act by interfering with the formation of higher order protein complexes. In the past years, several microProteins have been identified in plants and animals that strongly influence biological processes. Due to their ability to act as dominant regulators in a targeted manner, microProteins have a high potential for biotechnological use. In this review, we present different ways in which microProteins are generated and we elaborate on techniques used to identify and characterize them. Finally, we give an outlook on possible applications in biotechnology.

## Introduction

MicroProteins are small proteins that contain only a single protein domain, often a protein–protein interaction (PPI) domain but lack other functional domains found in the larger proteins that they are related to. MicroProteins can either completely inactivate their targets by forming non-functional heterodimers or alter their biological function by engaging the target protein in novel protein complexes. These interactions can occur either via identical PPI domains (homotypic microProtein inhibition) or by non-identical but compatible PPI domains (heterotypic microProtein inhibition) [1–3].

The characteristics of microProteins are typified by the first identified microProtein, INHIBITOR OF DNA BINDING (Id) in animals. The Id protein is a 16 kDa small protein consisting of only a helix–loop–helix (HLH) domain. Id can disrupt functional basic helix–loop–helix (bHLH) homodimers by forming bHLH/HLH heterodimers. This regulation fine-tunes cell proliferation and cell differentiation underlying muscle development [4]. LITTLE ZIPPER (ZPR) proteins were the first microProteins characterized in plants [5, 6]. ZPR proteins contain a leucine zipper domain but lack other domains required for DNA binding and transcriptional activation. ZPR proteins thus function in analogy to Id-type proteins and physically interact with class III homeodomain-leucine zipper (HD-ZIPIII) transcription factors to control developmental processes such as stem cell maintenance in shoot apical meristem (SAM) formation and leaf development.

Several microProteins have been identified in animals and plants. To date, 22 plant-specific microProteins have been characterized, all of which regulate transcription factors via protein–protein interaction [1, 3, 7]. In animals, microProteins regulating non-transcription factor proteins have also been characterized. An example is the viral protein U (Vpu) microProtein, which negatively regulates the human K^+^ ion channel TASK1 by sequestering it into a non-functional complex [8]. Additionally, Vpu mediates the interaction between TASK1 and TrCP, a component of the SCF^TrCP^ E3 ubiquitin ligase complex that results in the degradation of TASK1 [8]. In plants, we have recently demonstrated that using a synthetic microProtein approach, it is possible to negatively interfere with the function of multi-domain proteins that are dependent on homodimerization or heterodimerization for full its function [9].

In the past years, the term “microProtein” or “microprotein” was used in different contexts to describe different types of small proteins. For instance, small proteins such as cyclotides or knottins were named microproteins [10]. Considering the protein sequences, the absence of recognizable protein domains and the missing relationship to larger, multi-domain proteins, these peptides are not classified as microProteins (with the capital P).

**Fig. 1:**
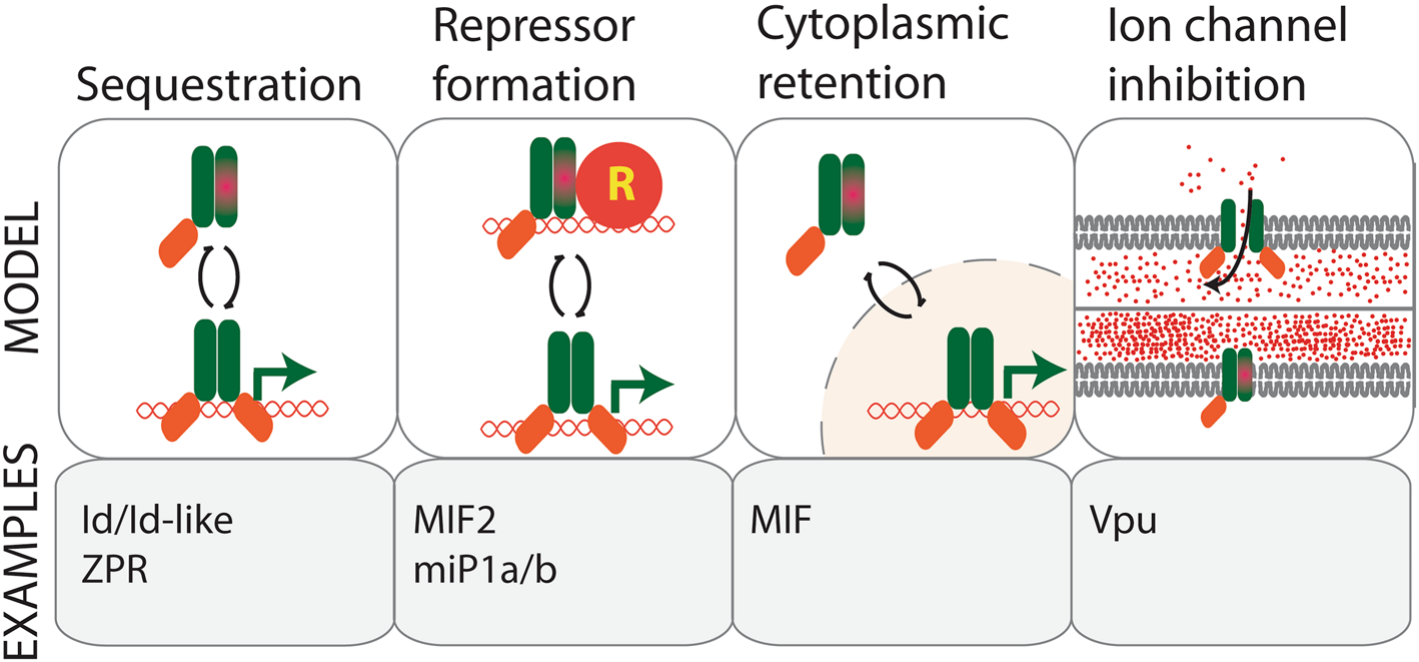
Different modes of microProtein regulation. MicroProteins can act by (1) sequestering their targets into non-functional complexes, (2) by attracting chromatin repressor proteins (R), (3) by sequestering the target in a subcellular compartment where it is inactive, (4) by interacting with ion channel subunits and compromising their transport capacity

The formation of non-functional homo/heterodimeric complexes is a primary mode of microProtein action; for example, the above described Id-like proteins and ZPRs are microProteins that function in this manner. Some microProteins are, however, capable of associating with higher order protein complexes, thereby increasing the functional diversity of their targets. An example of such microProtein mode of regulation has recently been discovered in the plant-specific miP1a and miP1b microProteins that regulate CONSTANS (CO), a positive regulator of flowering. MiP1a and miP1b exhibit classic microProtein characteristics such as a dominant negative phenotype and a homotypic PPI interaction with the target. Additionally, miP1a/b contain a carboxy-terminal PF(V/L)FL motif that facilitates interaction with TOPLESS (TPL) and TOPLESS-RELATED (TPR) co-repressor proteins. The interaction of miP1a/b with TPL bridges the interaction of TPL and CO, likely engaging CO in a repressor complex, which results in a late flowering phenotype due to failure to induce *FLOWERING LOCUS T* (*FT*) expression under inductive long day conditions. This reveals a novel mode of microProtein inhibition which involves the recruitment of co-repressors to change the activity of their targets (Fig. 1) [11].

Some microProteins can exert dual modes of inhibition. MINI ZINC FINGERs (MIFs) are a class of microProteins that exerts dual modes of inhibition in the regulation of floral architecture and leaf development. MIFs not only inhibit the target’s function by homotypic microProtein inhibition, but they also prevent their target from being nuclear localized by forming heterodimers with the target that results in cytoplasmic retention (Fig. 1) [12–14]. Furthermore, a recent study revealed that the MINI ZINC FINGER2 (MIF2) and the tomato homolog INHIBITOR OF MERISTEM ACTIVITY (SlIMA) interact with TOPLESS and HISTONE DEACETYLASE19 to repress target gene expression [15]. These findings point towards a possible role of transcription factor-related microProteins as adapters for chromatin regulators.

Depending on their mode of origin, microProteins can be classified as *trans*- or *cis*-microProteins. *Trans*-microProteins are individual transcription units that are evolutionarily related to larger genes encoding multi-domain proteins. There is evidence that some microProtein genes evolved in genome amplification events and subsequent domain-loss, resulting in single-domain-containing inhibitory small proteins [16]. *cis*-MicroProteins occur as a result of processes such as splicing, alternative translation start and stop site choices, which can give rise to mRNA isoforms encoding microProteins. In addition, microProteins may also be produced by post-translational processing, such as proteolytic cleavage which results in smaller products capable of interfering with their larger, un-cleaved precursor proteins.

**Fig. 2:**
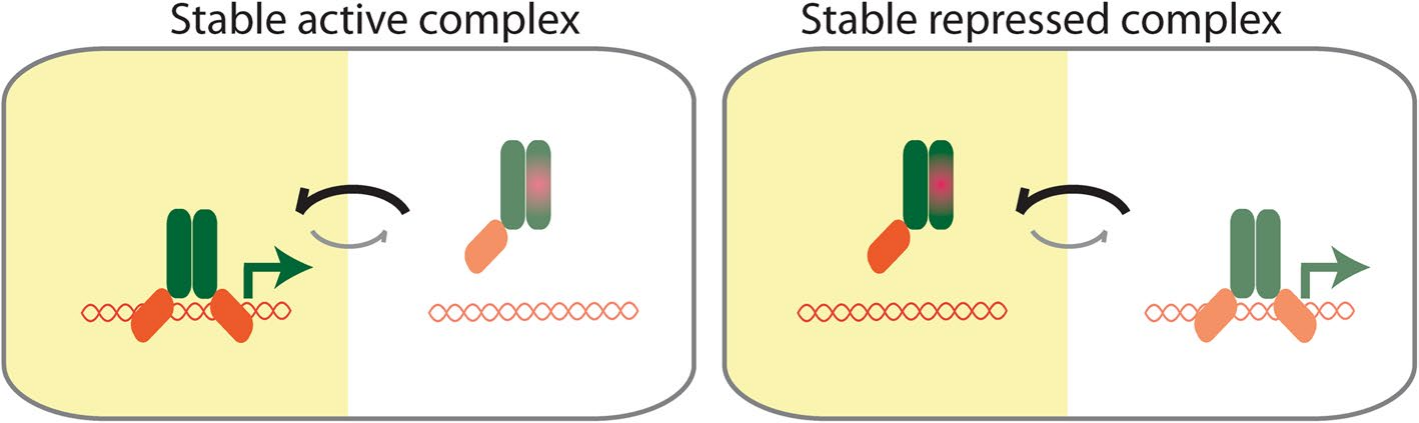
Two types of microProtein functions. MicroProtein interaction with its target and involved factors result in a stable repressed inactive form, here the system needs to be activated by certain factors to form an active complex. On the other hand, the target complex can be the active complex until the interaction of the microProtein with the target disturbs the stable target complex

MicroProteins are often described as negative regulators disrupting the normal stable state of protein complexes during physiological changes but this might not always be the case. For example, the LITTLE ZIPPER microProteins are transcriptionally controlled by HD-ZIPIII transcription factors, and subsequently, negatively regulate HD-ZIPIII protein activity by forming non-productive dimers. The state of the HD-ZIPIII as homodimeric proteins would be considered the normal state, whereas the HD-ZIPIII/ZPR heterodimer would be the inhibited state. For other transcription factors, however, it is conceivable that the microProtein-inhibited heterodimer is the prevalent form. Thus, in response to a physiological signal, the microProtein would disengage allowing the transcription factor to homodimerize and control gene expression. This would allow the system to remain in a repressed and inactive state until it is exposed to a condition or it reaches a stage when the system needs to be active such as certain developmental stages (Fig. 2).

The discovery and increasing biological importance of microProteins emphasizes the need for methods to identify novel microProteins involved in diverse processes. Here we present a framework that can be used to identify and study microProteins.

## Identification of microProteins using bioinformatics approaches

**Fig. 3:**
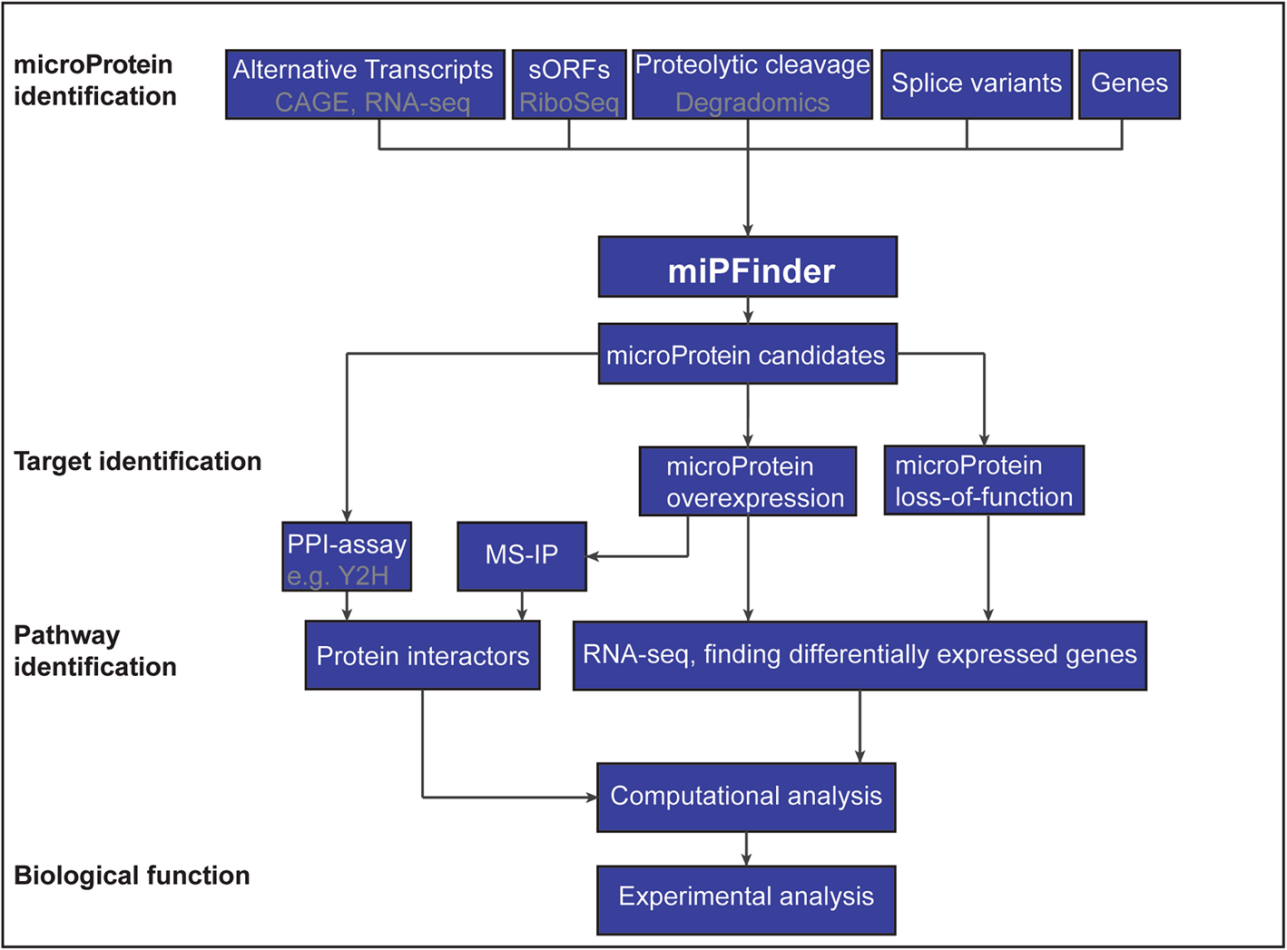
Flowchart of microProtein identification and characterization

The most straight forward method to identify microProteins is to analyze all annotated small proteins. All microProteins characterized to date, range in size from 7 to 20 kDa, which is roughly the size of a single protein domain. Although all microProteins are small proteins, not all small proteins are microProteins. There is a need to filter potential microProteins from small proteins using known characteristics of microProteins. MiPFinder is a recently published tool that utilizes information about protein size, domain organization, known protein interactions and evolutionary origin to identify microProteins and evaluate their potential to function as microProtein [17]. This computational approach can be applied to any complete or close-to-complete genome. While MiPFinder is a powerful tool in identifying novel microProteins, computationally detected candidates have to be evaluated experimentally to confirm their mode of action (Fig. 3).

MiPFinder is highly dependent on the quality of information stored in the respective databases, including gene annotations and definition of splice variants. For example, microProteins are known to be as small as 7 kDa and such small proteins may be overlooked in gene annotations making them unavailable for MiPFinder analysis [18]. In addition, not all protein–protein interactions can be predicted as of yet, and this problem, which also affects the discovery of novel microProteins, is gradually being addressed by the growing knowledge on protein interaction interfaces.

Detection of novel microProtein candidates also relies on information inferred from the properties of known microProteins. Up to date, most *bona fide* plant microProteins target transcription factors, therefore limiting our knowledge of the characteristics of microProteins to those observed in transcription factor regulation [1]. The discovery of more microProteins, especially in protein classes other than transcription factors will also improve the MiPFinder’s ability to identify novel microProteins.

MiPFinder is a valuable tool in identifying potential microProteins in fully or partially annotated genomes, however, even within fully annotated genomes certain protein products generated by alternative transcription or proteolytic processing cannot be entirely predicted as of yet and are therefore not annotated. In *Arabidopsis*, for example, although protein isoforms derived from alternative splicing are annotated, those generated from proteolytic cleavage or alternative transcription are difficult to predict because they are often produced in very specific physiological conditions. Experimental methods that aid in the identification of possible small protein products from such alternative processing have been developed, allowing for subsequent analysis using the MiPFinder.

## MicroProteins encoded by small open reading frames (sORFs)

Small open reading frames (sORFs) are frequently not annotated in genomes. Previous research showed that some sORFs play important roles in development [19] and some of these could potentially encode microProteins. Ribosome profiling (or ribosome sequencing, Ribo-seq) is a technique that is used to study a snapshot of transcripts that are translated (translatome) and proves instrumental in identifying sORFs [20]. By purifying either native ribosomes or using cell-type specific-tagged ribosomes, mRNAs that are in the process of being translated into proteins can be captured and sequenced. The selective sequencing of only ribosome-bound RNA is advantageous over other mRNA sequencing methods because the three-nucleotide periodicity allows conclusions to be drawn on the presence of the future protein. Ribo-seq also allows for the identification of translated unannotated and uncharacterized genes including those that are small in size (Fig. 3). Ribo-seq has already been used to detect novel uncharacterized proteins, many of which do not seem to originate from larger proteins and lack sequence similarity to annotated genes, thereby limiting the likelihood that they are microProteins [19, 21]. Small proteins such as CYREN and NoBody [22, 23] are examples of sORFs that are not microProteins because they lack certain microProtein characteristics such as a PPI domain or sequence relation to larger proteins. It is, however, possible that sORFs with a known PPI domain exist and function as microProteins. It is also possible that some sORFs with unknown sequence conservation could act as cryptic microProteins by folding into structures similar to PPI domains, allowing these sORFs to modulate target proteins. Using structural prediction, programs could aid in the identification of such sequence-unrelated microProtein-equivalents. Furthermore, studying evolutionary conservation of such sORF proteins could shed light on those that have been retained in the course of evolution and might therefore have biological relevance.

## Alternative transcripts can encode microProteins

*cis*-MicroProteins can be produced by alternative splicing, alternative transcription start and termination site usage. In addition to available information, alternative transcripts and transcription start/stop site analysis such as cap analysis of gene expression (CAGE), alternative transcription termination site isolation and RNA-Seq have been successfully used to monitor expression of alternative transcripts. CAGE identifies and quantifies transcription start sites using short sequence tags originating from the capped 5′ end of full-length messenger RNA [24]. Similar to CAGE, paired-end analysis of transcription start sites (PEAT) captures the capped ends of mRNA. PEAT has been used in *Arabidopsis* root samples to detect millions of transcription start sites including alternative ones, some of which might produce *cis*-microProteins [25]. Alternative transcription termination sites can be analyzed quantitatively by methods such as 3′ region extraction and deep sequencing (3′READS) that captures 3′ RNA polyadenylation regions with exceptional accuracy [26]. Quantification of transcript isoforms originating from alternative splicing can be assessed with RNA-Seq but requires either a well-annotated reference transcriptome or substantial effort and specialized software [27]. MicroProteins originating from alternative transcripts could be produced in response to a specific signal or be restricted to specific cell types. Identifying such alternative transcripts and correlating the resulting *cis*-microProtein candidates with a specific space or condition requires tailored bioinformatic analysis.

## Production of microProteins through proteolytic processing

Most known microProteins are protein products of transcriptional or post-transcriptional events [1]. However, microProteins can also be generated through post-translational processes such as proteolytic cleavage events (Fig. 3). Proteolytic cleavage is the specific hydrolysis of peptide bonds of a larger precursor protein by a protease, resulting in two or more shorter fragments; this process usually occurs in vivo and is irreversible. Proteolytic processing results in novel amino and carboxyl terminal functions referred to as neo-N and neo-C termini, respectively, which can be enriched before mass spectrometry (MS) analysis [28–30].

Proteolytic cleavage events resulting in protein products with microProtein-like dominant negative phenotype and inhibition of their precursor proteins, have been published. SERUM RESPONSE FACTOR (SRF), is a MADS box transcription factor that regulates cardiac development and function by binding to the serum response element (SRE). During Coxsackie virus infection, which leads to cardiomyopathy, the viral protease 2A, cleaves SRF to produce an N-terminal and a C-terminal fragment. The N-terminal cleavage product contains a DNA-binding domain but lacks the transactivation domain, which results in the dominant inhibition of its precursor SRF’s activity by competing for DNA binding without the activation capabilities [31]. A similar mechanism is observed during viral infection in neurodegenerative diseases. Viral protease 2A cleaves *trans*-active response DNA-binding protein-43 (TDP- 43), which is essential for the regulation of RNA metabolism. The N-terminal cleavage product acts as a dominant negative inhibitor by inhibiting the function of native, uncleaved TDP-43 in alternative RNA splicing [32]. These two examples show that proteolytic cleavage can produce products that can later act as microProteins.

Although many studies have been performed on the biological relevance of proteolytic cleavage events and the resulting products, the identification of the substrates and the cleavage sites of these events can prove challenging to study. Structural biology and enzymology are well established approaches that reveal valuable information on the activity of proteases. These approaches, however, are limited in the depth of information that can be provided such as the biological role of the protease activity on the substrate. Protease degradomics is a more recent approach that involves the application of genomics and proteomics in the identification of proteases and the substrates of proteolytic cleavage events [29, 33, 34]. Various methods in the area of degradomics such as PROTOMAP, COFRADIC, ATOMS and TAILS have been used to narrow down the complexity of protease-processed proteomes by providing a means to label and enrich for cleaved products. These methods utilize mass spectrometry (MS) to elucidate the protein variants produced by post-transcriptional, translational and post-translational events, allowing for the identification of multiple cleavage sites in a single experiment. Briefly, these methods involve in vivo or in vitro protease cleavage, blocking of the terminal ends depending on the terminal end of interest, and the MS analysis of the material.

Depending on the form in which the proteins are separated and analyzed by MS, the approaches can be further categorized into top-down and the bottom-up proteomics approaches. In top-down proteomics, intact protein products of proteolytic events can be analyzed using high-resolution MS [30]. This method is advantageous because it allows not only for the identification of proteolytic cleavage events but also for the preservation and monitoring of post-translational modifications (PTMs), mutations, and splice isoforms. The technique is, however, labor intensive and further complicated by the difficulty in labeling intact proteins and their higher potential for protein insolubility.

The bottom-up strategy is less time consuming and more widely used. This approach involves the digestion of the products of proteolytic events with another protease (work protease) prior to MS analysis. The MS analysis will therefore yield results based on small peptides which serve as surrogates to determine the precursor peptides. Although more widely used, this method is disadvantageous because it does not preserve information of PTMs and can result in difficulties in distinguishing isoforms and homologs [30, 35, 36]. Irrespective of what method is used to identify cleavage products, the resulting list of cleavage products can be assessed with the MiPFinder to determine if they qualify as potential microProteins.

## Functional characterization of microProteins

Novel microProtein candidates, identified with the computational program MiPFinder [17] or by alternative approaches, need to be experimentally validated to confirm their function (Fig. 3). The MiPFinder program can aid in the initial steps of characterizing potential microProteins by predicting possible targets and functions [17].

Unfortunately, in most cases, the direct target of microProtein candidates cannot be predicted, especially if the microProtein is either related to or part of a large protein family. Co-immunoprecipitation and MS can be used to identify microProtein complexes and provide further insight to their regulatory function [37]. The microProtein targets or the complex in which the microProtein candidate is part of, can be immunoprecipitated from total protein extracts; for example, protein extracts from transgenic plants overexpressing an epitope-tagged version of the respective microProtein candidate. The drawback of such MS analysis is the presence of a large number of non-specific or false-positive interactors. To overcome this problem, adequate controls must be used at different steps of the analysis; the presence of an additional tag can further aid in enriching for true interactors [38]. All potential interactors must also be carefully verified by further experiments under the appropriate physiological conditions.

To gain first insight into the function of a potential microProtein, ectopic expression of the microProtein from a strong constitutive promoter is often the method of choice [6, 11]. The phenotype of such gain-of-function overexpressors can give valuable insights on the physiological process that the microProtein is involved in. The phenotype of plants overexpressing a microProtein often resembles the loss-of-function mutant phenotype of the target and vice versa [1]. Therefore, it is important that the phenotype of the loss-of-function mutant is also determined.

In model plants, the likelihood of obtaining functional T-DNA knockout mutant plants for microProteins is reduced because of the small size of microProtein genes. Targeted approaches to generate knockdown or knockout mutants have been used successfully to generate loss-of-function mutants. MicroRNA-induced gene silencing (MIGS) fusion constructs have been employed to knockdown microProteins [11]. The disadvantage of this method is the incomplete loss of protein function, which may result in mild loss-of-function phenotypes; there is a chance that off-targets would be silenced in the process. Recent genome engineering systems such as zinc-finger nucleases (ZNFs), transcription activator-like effector nucleases (TALEN) and CRISPR-Cas9 have been successfully used in plant and animal genome engineering [39–42]. These approaches provide new and precise tools to generate targeted loss-of-function mutants of microProteins and their targets.

The above-mentioned methods are general tools to investigate the biological role of respective microProteins. Depending on the specific function of the microProtein and the target, other more targeted experiments may be needed for their in-depth characterization.

## Biotechnological relevance of microProteins: synthetic microProteins, new tools to control protein activity

The ever-growing interest in developing new crop traits such as stress tolerance, higher yield, and reduction of toxic compounds highlights the importance of biotechnological tools that can alter the development of plants to achieve these goals. In the past years, different methods that alter plant development have been used, such as the knockout or the ectopic expression of plant genes. Besides the use of loss-of-function or gain-of-function mutants, RNA interference (RNAi) has also been successfully used to generate new traits in crop plants [43]. However, the above-mentioned biotechnological tools have some drawbacks, for example, the knocking out of genes results in a permanent loss of gene-specific functions. Additionally, the use of RNAi in biotechnology can cause some unwanted effects such as off-targets effects or the instability of new traits through subsequent generations [43].

Another approach is the use of artificially derived microProteins known as synthetic microProteins. Here, a single functional domain of a multi-domain protein that is capable of interacting with its target protein is expressed in a controlled manner to obtain the desired effects. In Arabidopsis, the overexpression of the PPI domain of the transcription factors SUPPRESSOR OF OVEREXPRESSOR OF CONSTANS 1 (SOC1), AGAMOUS (AG) and LATE ELONGATED HYPOCOTYL (LHY) resulted in phenotypes similar to the loss-of-function mutant of the respective transcription factors. This was as a result of the ability of these PPI domains to heterodimerize with their source transcription factors and negatively regulate their function. This approach was also effective when the PPI domain of SOC1 was overexpressed in *Brachypodium distachyon* resulting in delayed heading [44]. Synthetic microProteins have also been used to successfully modulate flowering time of rice grown in long day conditions [45]. These studies reveal that the ectopic expression of the PPI domain of transcription factors can function as microProteins due to their ability to negatively regulate the larger transcription factors. A recent publication showed that synthetic microProteins are capable of regulating larger multi-domain proteins that are not transcription factor proteins [9].

Designing synthetic microProteins to negatively regulate larger proteins or to disturb protein complexes can lead to a more specific or controlled effect in comparison to the loss-of-function mutant. Furthermore, the expression of the synthetic microProteins can be regulated by different promoters, such as tissue-specific promoters or environmentally controlled promoters, making it possible to fine-tune the microProtein expression to achieve the desired effects. This emphasizes the enormous potential of synthetic microProteins to improve crops in the future. The limitation of this approach lies in the ability of synthetic microProteins to only regulate proteins with a compatible PPI domain.

## Conclusion

MicroProteins are small proteins containing only a single protein domain similar to or compatible with larger proteins. Most of the so far identified microProteins are characterized as negative regulators of their targets. They sequester their targets into non-functional dimers through protein interaction. This review shows that the microProtein mode of action is not limited to negative regulation. MicroProteins can have dual functions or function in higher order complexes, where other proteins are recruited to actively repress the target protein activity. Furthermore, it is conceivable that for some microProteins, the non-productive microProtein/target complex is the prevalent form, and this form can dissociate in response to certain physiological conditions, thereby releasing the microProtein target.

This review gives an overview of the different ways to identify novel microProteins and their potential targets. The MiPFinder program can identify potential microProteins, and their targets. The limiting factor of the MiPFinder is that it is based on annotated genes and sequenced genomes. Some small proteins are not annotated in the genome or generated by post-translational modifications such as proteolytic processing or alternative transcription. Those small proteins can be identified using methods such as degradomics or RiboSeq; they can be further verified using MiPFinder. All identified microProtein candidates, their targets and the physiological processes that they regulate need to be experimentally validated.

So far, all known microProteins regulate transcription factors, but the MiPFinder program and synthetic microProtein approaches in plants reveal that microProteins are capable of altering the function of a wider range of multi-domain proteins. Despite the first microProtein being discovered in mouse, functional studies of other animal microProteins are still lacking. Given the high percentage of human microProtein candidates associated with diseases [17], it is rather thought-provoking that microProteins have not received more attention in the biomedical field.
